# Letter to The Editor

**DOI:** 10.1155/1989/72130

**Published:** 1989

**Authors:** J. R. Goldring

**Affiliations:** 1 Department of Surgery Hairmyres Hospital East Kilbride Scotland; 2 University Department of Surgery Royal Infirmary of Edinburgh Laurieston Place Edinburgh EH3 9YW Scotland


*HPB Surgery* 1989, Vol. 1, pp. 257–258
Reprints available directly from the publisher
Photocopying permitted by license only

© 1989 Harwood Academic Publishers GmbH
Printed in the United Kingdom

### LETTER TO THE EDITOR

The Editor,
HPB Surgery

Dear Sir,

We read with interest the paper by Anderson and colleagues entitled "Haemoperitoneum after spontaneous rupture of liver tumor: results of surgical treatment". *HPB Surgery* 1988:**1**:81–83. We sympathise with their difficulty in diagnosis of this rare entity as reflected by our own anecdotal experience of a patient recently admitted to Hairmyres Hospital, East Kilbride, although in this case the approach to management necessarily differed.

A 67-year old man presented with sudden onset of epigastric and right upper quadrant pain which worsened and became pleuritic, spreading down the right flank and accompanied by nausea. Although he was hormotensive on admission his systolic pressure decreased as he developed signs of upper abdominal peritonitis. Of relevance in his previous medical history was a duodenal ulcer of seven years duration which had been well controlled on maintenance therapy, insertion of an aorto-bifemoral graft, and a left hemicolectomy for a Dukes' C leimyosarcoma of the sigmoid colon 12 months previously. A presumptive diagnosis of either a perforated duodenal ulcer or gangrennous cholecystitis was made.

At emergency laparotomy, a little free blood in the peritoneal cavity and dense adhesions were found with a normal gallbladder and a scarred but otherwise intact duodenum. Dividing adhesions around the liver, suddenly released a large quantity of fresh and clotted blood from the subphrenic space; 1000mls were aspirated. The source of the bleeding was identified as a ruptured solitary 12cm tumour in the dome of the right hemi-liver. Rather than attempt a hemi-hepatectomy in view of the patient's past medical history and the dense adhesions, his condition rapidly stabilized with initial control of the bleeding by packing and intravenous fluids. Fragments of the ruptured lesion were biopsied with drainage of the subphrenic space and lasting hemostasis was achieved by an oxycell gauze over the raw area. Post-operatively, he was electively ventilated for 24-hours and discharged 8 days later. Histologically, the tumour proved to be metastatic leiomyosarcoma.

Thus our own case highlights the difficulty in diagnosis encountered, though we were fortunated in that the bleeding was probably limited initially by the presence of adhesions and further controlled by simple haemostatic measures without having to undertake a major liver resection.

Yours sincerely

J.R. Goldring
Department of Surgery,
Hairmyres Hospital,
East Kilbride,
Scotland.

* Present address and address for correspondence:
University Department of Surgery,
Royal Infirmary of Edinburgh,
Laurieston Place,
Edinburgh EH3 9YW

